# Appreciations from the Lay Press

**Published:** 1920-05-08

**Authors:** 


### Appreciations from the Lay Press.

The following article by Mr. T. P. O'Connor, M.P., appeared in the *Daily Telegraph* of April 30 :—

We regret to announce the death of Sir Henry Burdett, who passed away yesterday evening. He was taken ill last February, as the result of overwork, and for some time hopes were entertained of his ultimate recovery, but the improvement in his condition was not maintained, and the end came owing to heart failure.

Sir Henry Burdett was a man who played many parts in life, and all energetically. There was a certain intensity in his nature which made him act in everything he undertook with a suggestion of a machine that was at once swift, relentless, restless, and a little noisy. He belonged to that class—the Lord Chief Justice used to be one of them—who take from sleep and give to work many of the hours that are necessary to the health of other men. Though he always appeared tranquil but vivacious, he seemed to be possessed by a perfect fever for work—a man who regarded life as a race with time; with time always hurrying after and threatening to draw level with, and even go beyond the work, which, I dare say, in a world where so many people are sluggards, produced now and then a good deal of hostility. The world gets the credit of always loving the lover; it does not always love the worker.

He came, like so many other celebrities, from the country parsonage; his father was a Leicestershire clergyman; his mother belonged to a county family in Lincolnshire, and he was born in 1847. I do not know anything of his earlier life; when I knew him first he was a great City official, secretary of the Share and Loan Department of the Stock Exchange. Associated with this office was his yearly production of a stately and bulky volume known as "Burdett's Official Intelligence of British, American, and Foreign Securities." It used to be one of the books which everybody associated with the City was bound to possess, for within its pages, in beautiful order, well tabulated, well printed, were particulars of all the public companies and other enterprises of the world; it was a sort of City Bible. Sir Henry also published innumerable other volumes on commercial questions : on the National Debt, on Sinking Funds, on Local Taxation; in fact, there seemed to be nothing in the high finance of the whole world which this remarkable mind could not set forth with lucidity, and with a grasp of countless statistics. If you had not met him before and were left to form your impressions of the man from his works, you might have considered him as one of those rigid-faced and stony-eyed men whose blood had turned to frigid ink in the consideration of the abstruseness of the financial problems and over the lifeless figures of the world's finance.

### Hospital Problems.

When, therefore, you met a man with an alert face, a friendly and almost caressing manner, with vividness in every movement, you received something of a surprise; and soon you found that his interests were not confined to mere figures and sovereigns, but that he had profound sympathy with almost everything that interested mankind; that he was full to overflowing with schemes for righting all kinds of abuses and follies; above all, that there was one subject which never ceased to fascinate and eventually to absorb him—namely, the public hospital. This was the reason probably why he founded and edited The Hospital, a journal entirely devoted to the subject which suggested its name. It would be difficult to follow all the ramifications which hospital work covers; but there was not one of these ramifications which Sir Henry Burdett did not pursue to the very end of its last turning. It was almost a passion with him to produce the result of his investigations in a memorandum; you had only to display a polite interest in some question that appealed to him to receive within a day or two a vast memorandum, dictated to a secretary in well-ordered sentences, with statistics set forth and side headings, and all the marks of a document emanating not from a private individual, but a great and public department with innumerable clerks on good salaries.

Pamphlet after pamphlet, book after book, appeared at intervals from this indefatigable and ceaseless worker—produced, some of them, in the hours when most people are in bed; he had habits, when I knew him best, which bear some resemblance not only to those of the Chief Justice, whom I have already mentioned, but to those of M. Clemenceau and Lord Haldane and other men, who think they can do their work best if they be awake and at their desks when all the rest of the world is still sleeping. These various volumes of his later life deal with all the hospital problems. He was intensely interested in everything that concerned that new race of nurses, unknown, it is sometimes forgotten, till Dickens caricatured them out of their old existence and Florence Nightingale led them into the new. He wrote on the food, the work, the recreation of nurses; elaborated a scheme for securing them old-age pensions; was active in procuring legislation for their registration; and suggested in another volume new methods for training in their profession.

The old-age pensions scheme, which ultimately took shape, had many pioneers, and Sir Henry, with his instinct for systematising philanthropy, was one of those who could be counted among the large army of original and constructive thinkers on the subject. He was, of course, especially interested in the subject where it touched the nursing sisterhood, with which he had been always so sympathetic. The appearance of Sir Henry Burdett was indicative of those characteristics which I have tried to set forth. He was tall, well built, with a finely-proportioned figure; the features were handsome and regular; the eyes had something of the blaze that denoted the ardent and restless temperament. A great and vivifying force in many good causes which demand the combination of keen business sense and strong sympathy with humanity is removed with his death.

---

### A Personal Recollection.

The editor of *Truth*, in the issue of Wednesday last, May 5, wrote the following personal recollection, which incidentally indicates the tribute of one editor to another:—

Sir Henry Burdett was, I should think, one of the hardest workers that ever lived. He had a great faculty for organising and stimulating the work of others, but he never spared himself, and the amount which he got through was prodigious. To this he largely owed his great success in life, which really began when, from being the superintendent of the Seamen's Hospital, he was appointed to the secretaryship of the Share and Loan Department of the Stock Exchange. By his energy and ability he brought that office to a degree of efficiency and usefulness which it had never before possessed, and his famous compilation, "Burdett's Official Intelligence," was a monument of well-directed energy, as well as a mine of invaluable information for "the City." His work on the Stock Exchange earned for him the confidence and, in many cases, the friendship of all the magnates of the City, which, in turn, helped him enormously in the philanthropic work with which he became more and more identified as time went on.

It would be difficult to overstate the value of the work which Sir Henry Burdett did for the hospitals of this and other countries—the work which he made his own and for which he deserves to be gratefully remembered. There has been a revolution in hospital administration during the last thirty or forty years, and the credit for all that has been gained thereby is largely due to Sir Henry Burdett. He brought to bear upon the subject a practical knowledge, acquired by his early administrative work in Birmingham and at Greenwich, informed by medical study (which he always kept up), and enlarged by frequent travelling in all parts of the world. Many years ago he told me that he had visited nearly every hospital in Europe and North America and many of those in other parts of the globe; and his reputation as an authority on this subject was as high in foreign countries as here. By writing and lecturing, and the influence which he exerted in many ways, he effected innumerable reforms, and he may be truly said to have standardised hospital administration throughout this country.

The mere amount of money which Sir Henry Burdett raised for hospital work was prodigious. He was really the originator of the King's Hospital Fund for London (originally founded by his late Majesty as the Prince of Wales's Hospital Fund), and the League of Mercy was not only conceived by him, but owed its original success to his unsparing energy in organising and starting it. Another of his works, on which he justly prided himself, was the foundation of a pension fund for nurses. With all the work which he devoted to objects of this kind, he found time to dabble in politics, and interest himself actively in all sorts of public questions, besides editing and contributing largely to The Hospital, which he founded and practically owned. He really had an instinct for journalism, and within the last few years he had started *The Nursing Mirror*, which has become a most successful and useful little journal.

Like most men of energy who achieve anything of note in the world, Sir Henry Burdett did not make friends everywhere. He was very much the fighting man, confident in his own opinions, and not always discreet in speech. I have heard very unkind things said about him, though few which I could not contradict from my own knowledge. He had the good fortune to win the favour of so excellent a judge of men as King Edward, to whom he probably owed the K.C.B., which he received in 1897, and certainly the K.C.V.O., which was bestowed on him in 1908. In private life Sir Henry was generous, hospitable, and always the best of company, and he was as unsparing of himself in the service of his friends as he was in the public work of his active and useful life.

---

### "Birmingham Post,"

*May* 1, 1920.

Sir Henry Burdett while in Birmingham was for a time secretary to the society which had for its aim the exemption of charities from rating, a matter for which he worked hard. The training of nurses was also a subject which appealed to him, and he was the first to organise a system for the training of nurses according to modern ideas and methods. As a young man he studied political organisation, and he and the late Mr. Joseph Chamberlain were sometimes to be found speaking together before the Edgbaston Debating Society, of which he was then treasurer. This was in the years preceding Mr. Forster's Education Act, and Mr. Burdett, as he then was, co-operated in the campaign to promote Bible reading in board schools, with the result that a majority was obtained in the first School Board for Birmingham, who carried out this programme.

